# Expression Analysis of XTH in Stem Swelling of Stem Mustard and Selection of Reference Genes

**DOI:** 10.3390/genes11010113

**Published:** 2020-01-20

**Authors:** Mengyao Li, Fangjie Xie, Qi He, Jie Li, Jiali Liu, Bo Sun, Ya Luo, Yong Zhang, Qing Chen, Fen Zhang, Ronggao Gong, Yan Wang, Xiaorong Wang, Haoru Tang

**Affiliations:** 1 College of Horticulture, Sichuan Agricultural University, Chengdu 611130, China; limy@sicau.edu.cn (M.L.); m17778649365@163.com (F.X.); liujiali1127@163.com (J.L.); 14099@sicau.edu.cn (B.S.); luoya945@163.com (Y.L.); zhyong@sicau.edu.cn (Y.Z.); supnovel@gmail.com (Q.C.); zhangf_12@163.com (F.Z.); wangyanwxy@163.com (Y.W.); wangxr@sicau.edu.cn (X.W.); 2 Institute of Pomology and Olericulture, Sichuan Agricultural University, Chengdu 611130, China

**Keywords:** stem mustard, reference gene, qPCR, *Brassica juncea*, *XTH* genes

## Abstract

Accurate analysis of gene expression requires selection of appropriate reference genes. In this study, we report analysis of eight candidate reference genes (*ACTIN*, *UBQ*, *EF-1α*, *UBC*, *IF-4α*, *TUB*, *PP2A,* and *HIS*), which were screened from the genome and transcriptome data in *Brassica juncea*. Four statistical analysis softwares geNorm, NormFinder, BestKeeper, and RefFinder were used to test the reliability and stability of gene expression of the reference genes. To further validate the stability of reference genes, the expression levels of two CYCD3 genes (*BjuB045330* and *BjuA003219*) were studied. In addition, all genes in the xyloglucan endotransglucosylase/hydrolase (XTH) family were identified in *B. juncea* and their patterns at different periods of stem enlargement were analyzed. Results indicated that *UBC* and *TUB* genes showed stable levels of expression and are recommended for future research. In addition, *XTH* genes were involved in regulation of stem enlargement expression. These results provide new insights for future research aiming at exploring important functional genes, their expression patterns and regulatory mechanisms for mustard development.

## 1. Introduction

Quantitative real-time PCR (qPCR) is considered an important method for detection and analysis of levels of gene expression. It has many advantages such as high accuracy, specificity, low cost, and reproducibility [1]. However, the accuracy of qPCR results is influenced by the quality of RNA, efficiency of reverse transcription, primer specificity, sample volume, and amplification efficiency [2]. In order to improve this accuracy, it is important to introduce one or more reference genes for standard correction expression. Reference genes are those that are expressed at all times for maintenance of the basic life activities of a cell, and their expression levels are less affected by the environmental factors. In plants, a number of reference genes have been identified including *ACTIN*, *PP2A*, and *TUB* and are now commonly used in gene expression analyses [3]. These genes are mainly involved in maintaining basic cellular functions such as cell structure and primary metabolism.

In recent years, there have been many studies suggesting that stable expression of reference genes varies with experimental conditions [4]. For example, *ACTIN* exhibits different expression patterns in different plants, tissues, and experimental conditions [5]. Study in *Cannabis* showed that *UBQ* was the most stable gene in different leaf samples, while *PP2A* was the most stable reference gene in different organs [6]. In garlic, *UBQ* and *ACTIN* are the most reliable reference genes and therefore recommended for analysis of different developmental stages and abiotic stress management, respectively [7]. *UBQ* gene was the most stable reference gene in sugarcane leaves under drought stress, while *PP2A* was the best reference gene under sorghum *Mosaic virus* [8]. Moreover, in *Sorghum*, *PP2A* was found to be the most stable gene in analysis of abiotic stress, while *UBC* showed the least stability [9]. Since reference genes do not always show a complete stable expression in response to various conditions or across species, their reassessment under certain conditions is crucial to validating accuracy during the calculated results of gene expression studies.

Plant growth and development involves cell wall loosening and remodeling. Cell wall loosening is the basis for rapid cell enlargement while cellulose-hemicellulose networks play a leading role in cell wall remodeling. This process is regulated by xyloglucan endotransglucosylase/hydrolase (XTH), an enzyme involved in root growth [10], stem elongation [11], flower development [12], as well as promoting fruit ripening [13]. A study of *XTH* genes in *B. juncea* showed that *BjXTH1* and *BjXTH2* could function in cell expansion of the pith tissue [14]. In addition, XTH also plays an important role in response to stresses. Overexpressing a pepper XTH gene *CaXTH3* in *Arabidopsis* and tomato plants was found to confer high resistance to drought and salt stress in transgenic plants [15,16]. A *PeXTH* gene from *Populus euphratica* was also observed to mediate plant responses to salt stress in transgenic tobacco [17]. 

Stem mustard (*Brassica juncea* var. *Tumida* Tsen et Lee) is a variety of mustard and one of the unique vegetable crops in China. It belongs to the Cruciferous family and its tumor stems are bulged and protruded. Currently, studies on stem mustard are mainly focused on breeding, cultivation, and nutrient quality. There is a need for studying regulatory networks and molecular mechanisms of stem swelling targeting appropriate reference genes in different development stages of stem mustard. This is because of the importance of this trait in yield determination. In this study, we selected eight relatively stable reference genes from genome and transcriptome databases, and evaluated their expression levels in all samples during the process of stem development. We used four statistical softwares geNorm, NormFinder, BestKeeper, and RefFinder with two target genes, *BjuB045330* and *BjuA003219*, that encode CYCD3 proteins for verification of the constant levels of the selected reference genes. All *XTH* genes were identified and screened in the *B. juncea* genome to understand their roles in stem swelling. The findings of this study will facilitate to future studies in mechanisms of development of stem mustard.

## 2. Materials and Methods

### 2.1. Plant RNA Extraction and cDNA Synthesis

**Figure 1:**
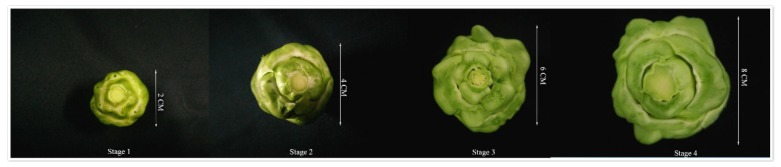
Growth state of stem mustard in four different development stages.

The stem mustard variety “Fuza No.2” was planted in the teaching base of Chongzhou, Sichuan Agricultural University. Four stages of stem in stem mustard were selected: the diameter of stem in the stage 1 was 2 cm (S1), the diameter of stem in stage 2 was 4 cm (S2), the diameter of stem in stage 3 was 6 cm (S3), and the diameter of stem in stage 4 was 8 cm (S4) (Figure 1). Three biological replicates were set for each sample. 

Total RNA was extracted from plant materials using the TransGene kit (Beijing, China) according to the manufacturer’s instructions. Quality and purity of the RNA were determined using a NanoDrop ND 2000 spectrophotometer (ThermoFischer, Waltham, MA, USA) and agarose gel electrophoresis. DNA contamination was eliminated from the RNA by DNAseI treatment, followed by synthesis of complementary DNA (cDNA) using the TransGene reverse transcription kits (Beijing, China).

### 2.2. Selection of Reference Genes, Primer Design, and Cloning

Eight candidate reference genes (*ACTIN*, *UBQ*, *EF-1α*, *UBC*, *IF-4α*, *TUB*, *PP2A,* and *HIS*) were selected from the whole-genome and transcriptome data based on homologous similarity in other plant species. Primers were designed using Primer Premier 6.0 software and are outlined in Table S1. The normal amplification system as follows: template cDNA: 1 µL, 2 µL of each primer (0.2 μM), 2.5 mm dNTPs, 10 × *EasyTaq* buffer 5 µL, *EasyTaq* enzyme 0.5 µL, ddH_2_O 37.5 µL, total 50 µL. PCR was carried out then amplification products confirmed using a 1% agarose gel electrophoresis. The products were cloned into the *pEASY*-T1 vector, and sent to TransGene (Beijing, China) for sequencing.

### 2.3. Real-Time Quantitative PCR Amplification (qPCR)

The qPCR was performed on BIO-RAD CFX96 quantitative PCR instrument (BIO-RAD, Hercules, CA, USA) with SYBR Premix *Ex Taq* (TransGene, Beijing, China). The 18-fold dilution of cDNA was used as the template to conduct qPCR for each gene. The standard curve of qPCR was performed using a 10-fold dilution series (10^×^, 10^2×^, 10^3×^, 10^4×^, 10^5×^) of non-experimental treated cDNA as a template. Each sample was repeated three times. Each reaction system was 20 µL, including 10 µL SYBR Green I mix, 0.4 µL each primer, 2.0 µL of diluted cDNA, and 7.2 µL of ddH_2_O. The PCR conditions are as follows: at 95 °C for 30 s for pre-denaturation, 40 cycles of denaturation at 95 °C for 5 s, annealing at 60 °C and extension for 30 s. A melting curve was analyzed from 65 °C to 95 °C to verify the primer specificity. Each 96-well plate contained a standard curve system and a cDNA-free system. The primer amplification efficiency of each candidate reference gene is expressed by the slope of the linear regression model: E% = (−1 + 10 [−1/slope]) × 100%.

### 2.4. Data Analysis and Evaluation of Stability of Reference Genes

The Cq value of each sample was obtained following qPCR. Three analysis software geNorm [18], NormFinder [19] and BestKeeper [20] were used to evaluate the stability of the reference genes, while RefFinder [21] was used to calculate the comprehensive ranking. Based on these analyses, the most moderately and the least stably expressed genes were selected as the standardized factors.

### 2.5. Identification of XTH Family Genes in B. juncea

We used XTH sequences in *Arabidopsis thaliana* from TAIR (https://www.arabidopsis.org/) as queries in BLAST to identify and retrieve *BjuXTH* genes in whole-genome sequence of *B. juncea* from the Brassica database website (BRAD) (http://brassicadb.org/brad/). BLAST analysis was carried out using default parameters. Candidate XTH proteins were further submitted to the Pfam and NCBI databases for verification of the structural domains. A multiple sequence alignment of XTH proteins from *B. juncea* and *Arabidopsis* was performed using Clustal W (http://clustalw.ddbj.nig.ac.jp/), followed by phylogenetic analysis using MEGA 5.0 (https://www.mega.com/). 

### 2.6. Expression Analysis and Validation of XTH Using qPCR

Analysis of the stem mustard variety “Fuza No.2” transcriptome at different development stages (S1, S2, S3, and S4, respectively) was carried out. The data obtained by sequencing has been submitted to NCBI website, and the accession number is SRP151320. FPKM values used to reveal expression abundance of *XTH* genes. Nine differentially expressed *XTH* genes were selected from three subgroups. A qPCR analysis was then performed to validate the observed expression profiles of several *XTH* genes, with the best reference genes selected for normalization of gene expression. A trend line with a fitting coefficient *R*^2^ value closer to 1 was selected for regression analysis. The relative expression levels were calculated with the 2^−ΔΔC^^t^ method [22]. 

## 3. Results

### 3.1. Specificity and Efficiency in Amplification of Candidate Reference Genes

The stem mustard cultivar “Fuza No.2” (Figure 1) was studied with specific primers of eight candidate reference genes (outlined Table S1) used for RT-PCR amplification. The candidate reference genes were cloned for use in subsequent experiments (Figure S1). Sequence information was submitted to NCBI website, with accession numbers from MN566462 to MN566469.

**Figure 2:**
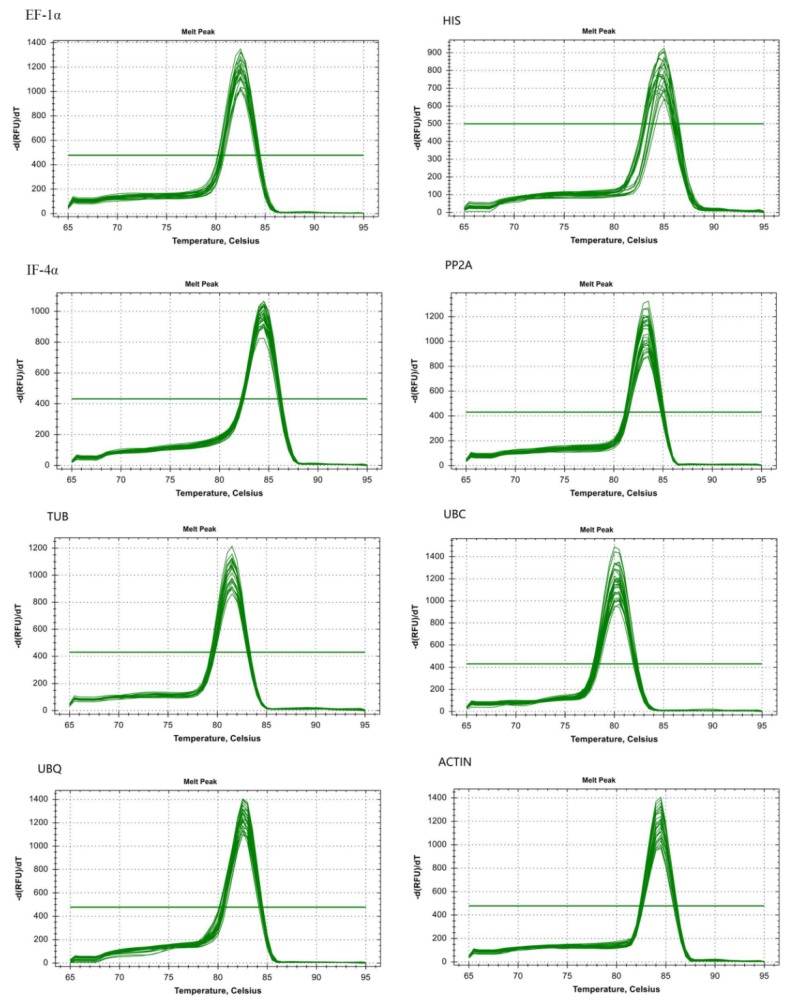
Melting curve analysis of eight candidate reference genes of stem mustard.

Eight genes were amplified by qPCR and resulted in products ranging from 100 to 267 bp (Table 1 and Figure S2). Analysis of melting curves for primer specificity resulted in a single peak with an expected amplification effect (Figure 2). A 10-fold cDNA dilution was used as the template for analysis of primer efficiency (*E*%) and coefficient of correlation (*R*^2^). We found the value of *E*% in the eight reference genes to be between 94.2 and 108.7%, and the *R*^2^ was above 0.99 (Table 1 and Figure S3). This showed that the result met the requirements of subsequent experimental analysis [23].

### 3.2. Evaluation of Expression Stability of Reference Genes

#### 3.2.1. Cq Value of Candidate Reference Genes

**Figure 3:**
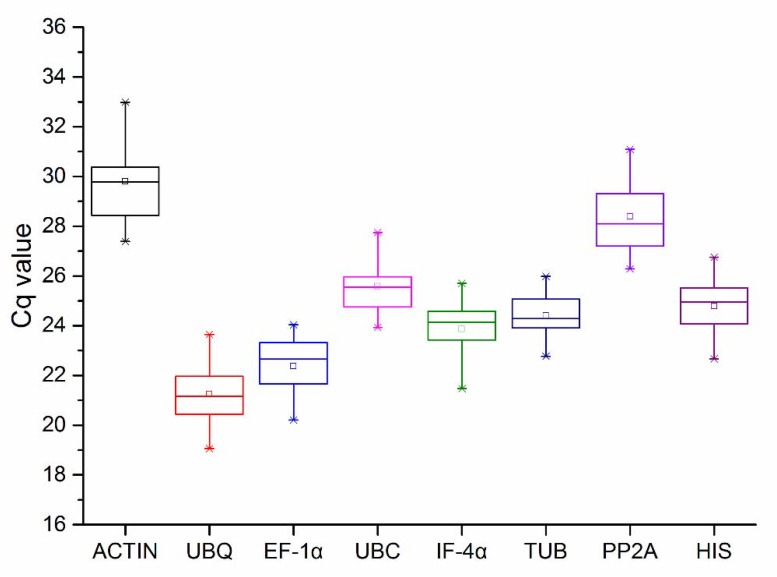
The Cq values of eight genes in all samples. The asterisk in the figure represents outliers, the line across the box represents median, the box represents a range of 25 to 75% in the value of Cq, and the extension lines at the top and bottom represent a range of 5 to 95%, with different levels of abundance for each reference gene transcript. Cq: quantification cycle.

The Cq value obtained after qPCR was used to represent gene expression levels. There were 36 Cq values for each reference gene across the four experimental treatments, three biological, and three technical replicates (Table S2). A wide range of Cq values was obtained across the eight genes. As shown in Figure 3 and Figure S4, all test samples were distributed between 19.05 (*UBQ*) and 32.98 (*ACTIN*), and the mean Cq values of all genes ranged from 21.30 (*UBQ*) to 29.53 (*ACTIN*). Distribution of Cq value for *TUB* was more concentrated than other genes, whereas that of *ACTIN* showed the biggest variation. Low Cq indicates high transcriptional expression level of the gene, while the converse is true for a high value. *UBQ* and *EF-1α* showed high transcriptional expression levels with low Cq, while *ACTIN* and *PP2A* resulted in low expression.

#### 3.2.2. geNorm Software Analysis

**Figure 4:**
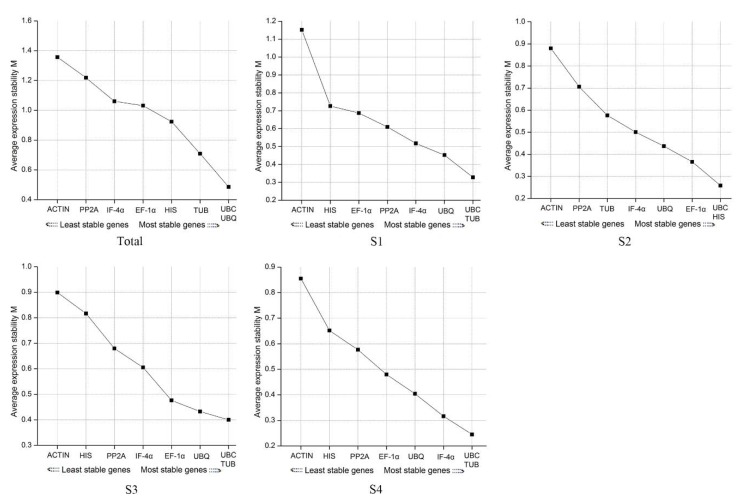
The *M*-values of the eight genes in different development stages. *M*: Average stability index.

geNorm compares constant levels of candidate reference genes by calculating the average stability index (*M*). A threshold value of *M* is set at 1.5, and serves as the criterion for determining whether gene expression is stable or not. Ideally, a stably expressed gene should have *M*-value below 1.5. The lower the value, the more stable the expression. *M* values of the eight genes at different development stages were all lower than 1.5, indicating that they were all stably expressed (Figure 4). However, the most stable genes at different stages were variable. In S1, S2, and S4, *UBC* and *TUB* were the most stable genes, while *UBC* and *HIS* were the most in S2. Overall analysis of the samples indicated that *UBC* and *UBQ* resulted in the most stable performance. The *UBC* had the lowest *M*-value across the four stages indicating that it was the best among all eight reference genes. *ACTIN* had the highest *M*-value in the four stages and was therefore the most unstable. 

#### 3.2.3. NormFinder Software Analysis

NormFinder converts a Ct value into relative expression levels (Q), and calculates the stability of reference genes according to the input program of Q value. This value was used to evaluate the stability of the reference genes followed by selection of the optimal reference gene. A small stability value indicates a more stable reference gene with a higher value pointing toward an unstable gene. *UBC* had the lowest stability value resulting in the most stable performance in S1 and S4, but it ranked second in S2 and S3. On the other hand, *IF-4α* and *EF-1α* ranked first in S2 and S3, but showed an unstable performance in the overall ranking (Table 2). *UBC* and *TUB* performed well in terms of stability during the four stages of development, while *ACTIN* was the lowest stability gene.

#### 3.2.4. BestKeeper Software Analysis

For BestKeeper, standard deviation (SD) and the coefficient of variation (CV) of Cq values were calculated to reveal the level of gene expression. Here, genes with small SD and CV values are considered more stable genes. In the overall ranking, expression of *TUB* and *UBC* genes was the most stable. We observed that *EF-1α* had the lowest SD and CV values in S1, S3, and S4 but ranked sixth in S2, suggesting that this gene exhibited the most stable levels of expression (Table 2). *UBC* is second in S3 and fourth in S4. *TUB* showed the best performance in S2 but was less so in the other three periods. Notably, we also found that *ACTIN* showed the lowest stability in S1, S3, and S4. Among all the groups, *TUB* and *EF-1α* displayed good performance, while *ACTIN* was the least stable gene in most groups. 

#### 3.2.5. RefFinder Software Analysis

**Figure 5:**
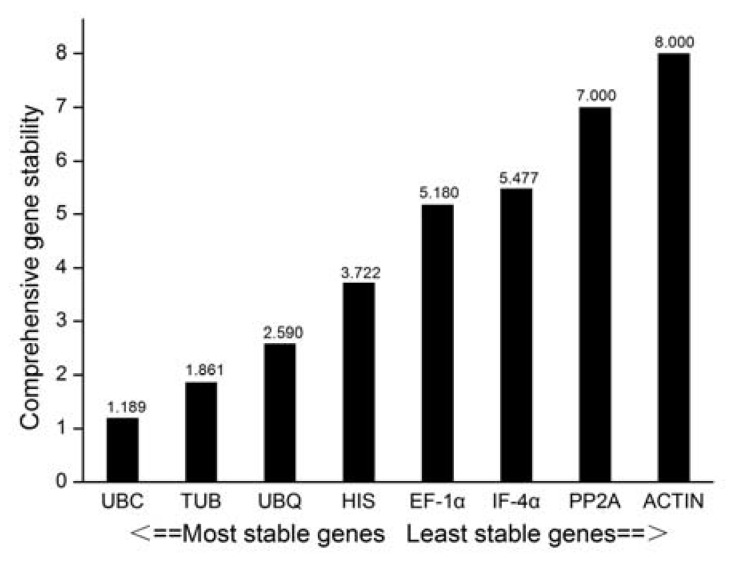
RefFinder comprehensively analyzed the expression stable level of eight candidate reference genes during different development stages of *B. juncea.*

RefFinder calculates the geometric mean for each gene based on geNorm, NormFinder, BestKeeper, and ΔCt to obtain a composite index ranking. The smaller the value, the more stable the gene expression is. We observed that *UBC* ranked first as the most stable gene, while *ACTIN* was the least stable. In general, the stability ranking followed the pattern; *UBC* > *TUB* > *UBQ* > *HIS* > *EF-1α* > *IF-4α* > *PP2A* > *ACTIN* (Figure 5). 

### 3.3. Determination of the Optimal Number of Reference Genes

**Figure 6:**
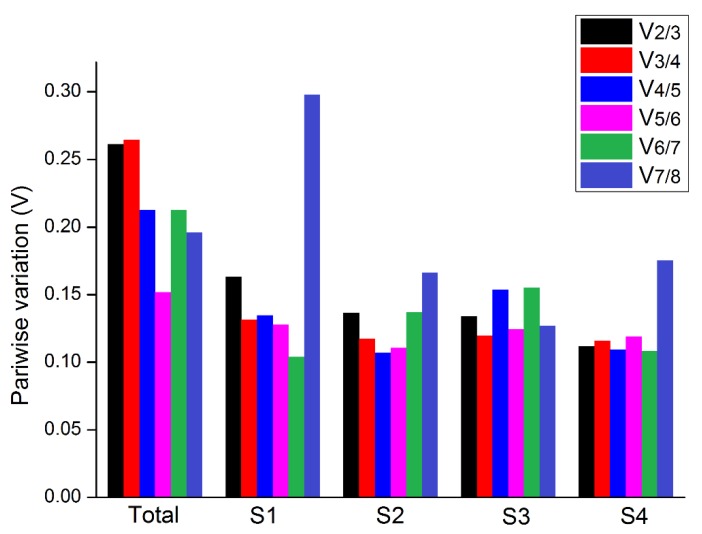
Pairwise comparative analysis of eight reference genes in *B. juncea.*

We used geNorm to calculate the paired difference value V_n_/_n+1_ and determine the optimal number of reference genes required in an experiment. When V_n_/V_n+1_ > 0.15, NF_n_ gene is not stable, and the NF_n+1_ gene therefore needs to be introduced. When V_n_/V_n+1_ is less than 0.15, the optimal number of reference genes is NF_n_. On the other hand, when V_n_/V_n+1_>0.15, it is necessary to introduce the NF_n+1_ gene. Addition of the third gene relatively improved the combination stability in S1. For S2, S3, and S4, only two genes were sufficient with the value of V_2_/V_3_ < 0.15 (Figure 6 and Table S3). When all samples were evaluated together, all V_n_/V_n+1_ values were higher than 0.15. However, although using more number of reference genes may help in reducing system deviation, it is not a necessary criterion [18].

### 3.4. Validation of Reference Genes Using qPCR 

**Figure 7:**
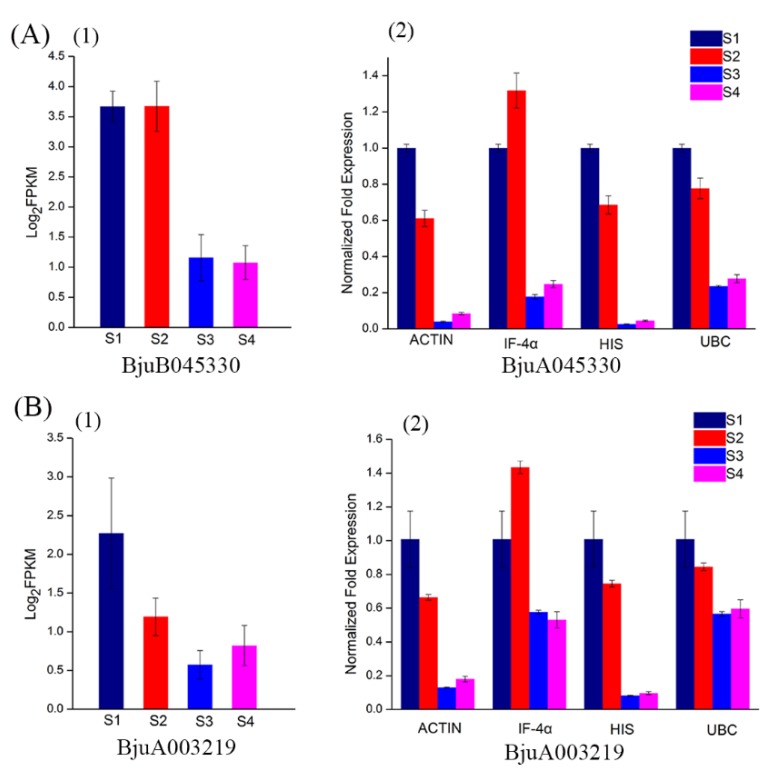
Transcript abundance and qPCR analysis of *BjuB045330* and *BjuA003219* at different stages of development. (**A**) Transcript abundance (**1**) and relative expression level (**2**) of *BjuB045330*. (**B**) Transcript abundance (**1**) and relative expression level (**2**) of *BjuA003219*. FPKM values were log2-based.

In order to further validate gene expression, a qPCR was conducted on four reference genes including the most stable (*UBC*), moderately stable (*HIS* and *IF-4α*), and least stable (*ACTIN*) genes. CYCD is a gene family related to cyclin in type D plants. Studies have proved that CYCD3 is involved in cell division, differentiation, and development of roots and fruits [24,25]. Two CYCD3 genes cloned from the stem mustard, resulted in varied expression levels in different parts of a tuberous stem [17]. Based on the transcriptome data, we found that two CYCD3 genes, *BjuB045330* and *BjuA003219*, showed high expression abundance at S1 and S2 and low abundance at S3 and S4 (Figure 7(A1,B1)). 

In this study, expression of *BjuB045330* and *BjuA003219* were calculated using the selected reference genes. Expression levels across the genes were significantly different. When normalized using the most stable gene, *UBC*, expression patterns of *BjuB045330* and *BjuA003219* were downregulated from S2 to S3, and maintained at a low level in S3 and S4. Similar expression patterns were generated using *HIS* and *ACTIN* although the levels of *BjuB045330* and *BjuA003219* were significantly low in S3 and S4 (Figure 7(A2,B2)). When *IF-4α* was used, expression levels of *BjuB045330* and *BjuA003219* showed a strong bias compared with the other three reference genes. In addition, it was found that there was a good concordance between qPCR and transcriptome data when normalized with *UBC*, indicating the reliability of selection of reference gene. These results not only validated the accuracy of reference genes, but also demonstrated that appropriate reference genes are very important for accurate analysis of expression of a target gene.

### 3.5. Identification and Phylogenetic Analysis of XTH Gene Family in B. juncea

**Figure 8:**
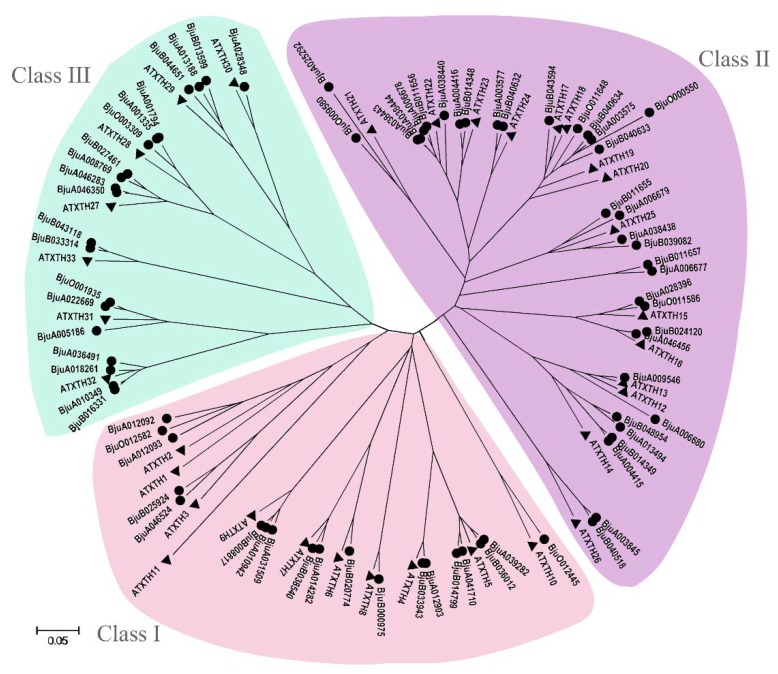
Phylogenetic tree of xyloglucan endotransglucosylase/hydrolase (XTHs) from *B. juncea* and *Arabidopsis*. The phylogenetic tree was constructed by the Neighbor-joining method with 1000 bootstrap replications. The three groups are represented with different colors.

To identify all the *XTH* genes in *B. juncea* genome, a basic local alignment search tool (BLAST) was performed using the *AtXTH* genes as queries and a total of 74 sequences of *BjuXTH* genes retrieved. To investigate the phylogenetic relationship of XTH members, 33 genes in *Arabidopsis* and the 74 from *B. juncea* were used to construct a phylogenetic tree (Figure 8). All the XTHs can be clearly divided into three subfamilies: Class I, Class II, and Class III. The largest class was Class II, having 35 members in *B. juncea* and 15 in *Arabidopsis*. Class I contained 19 XTH genes from *B. juncea*, whereas 11 were from *Arabidopsis*. Class III consisted of 20 and 7 XTH proteins in *B. juncea* and *Arabidopsis*, respectively.

### 3.6. Gene Expression Analysis of Mustard XTH Family

**Figure 9:**
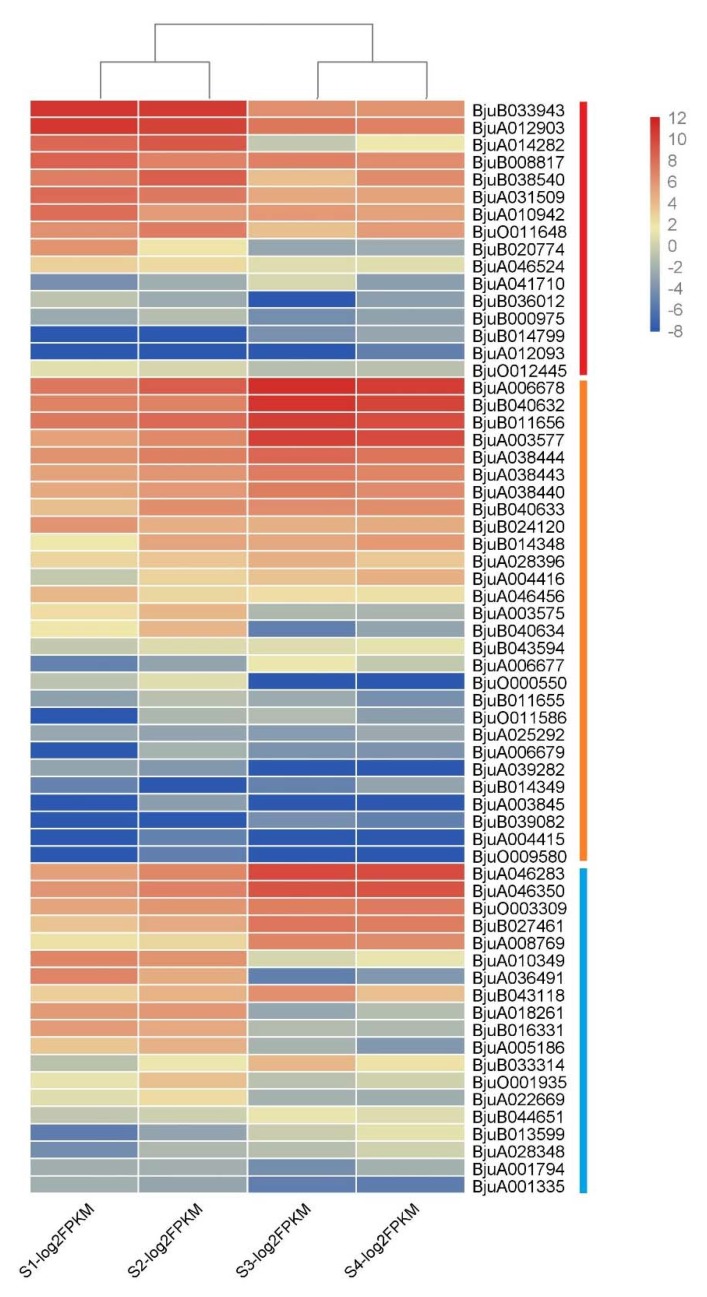
Expression levels of *BjuXTH* genes at different developmental stages in stem swelling. Fragments per kilobase per million (FPKM) values of *BjuXTH* genes were transformed by log2, and the heatmap was constructed with Multiexperiment Viewer software.

The expression profiles of 74 *XTH* genes were further analyzed based on the transcriptome data of four stages of stem development in stem mustard. Results showed that 63 genes were expressed in at least one stage while 11 were not detected. *XTH* genes showed differential abundances at various development stages. Particularly, most genes in Class I showed a high expression in S1 and S2, while the highest in S3 and S4 was observed in Class II and III genes (Figure 9). In Class I, *BjuB033943* and *BjuA012903* had a relatively high abundance. With regards to the Class II subgroup, expressions levels of *BjuA006678*, *BjuB040632*, *BjuB011656,* and *BjuA003577* were higher in S3 and S4 compared to S1 and S2. Most members of Class III especially *BjuA046283*, *BjuA046350*, *BjuO003309*, *BjuB027461,* and *BjuA00876* showed higher expression levels and these were significantly higher in S3 and S4. A large number of *XTH* family genes were highly expressed during the period of stem development, suggesting that XTH genes might play important roles in the development product organs of stem mustard.

**Figure 10:**
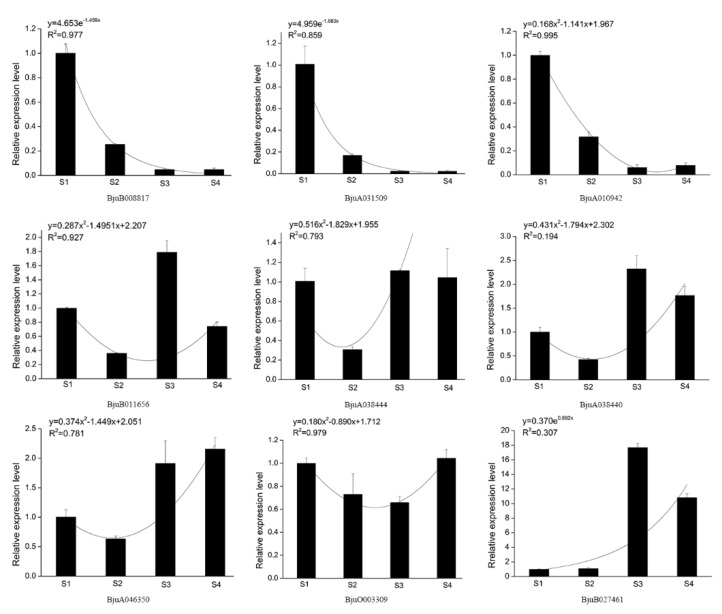
Gene expression and regression analysis of *BjuXTH* family genes.

Based on the transcript abundance across different development stages of stem in stem mustard, several XTH genes were targeted via qPCR to evaluate their expression levels during stem swelling. *UBC* was used as the reference gene for standardization of expression. Three genes, *BjuB008817*, *BjuA031509*, and *BjuA010942*, that belonged to Class I, reduced expression levels during stem swelling (Figure 10). Class II genes (*BjuB011656*, *BjuA038444,* and *BjuA038440*) resulted in variable expression patterns, with higher levels in S3 and S4. In Class III, the expression levels of *BjuA046350* and *BjuB027461* were increased from S2 to S3 and keep higher expression in S3 and S4. Overall, qPCR results for most XTH genes corroborated with the transcriptome data.

## 4. Discussion

The study on expression profiles of many genes is commonly performed relying on techniques like RNA-seq, microarray, Northern blot to reveal an underlying expression dynamic. However, results of gene expression data must then be validated to obtain reliable data that will support working hypotheses directed at a better understanding of development or environmental responsiveness [26]. The qPCR technology is an important tool for validating gene expression in various biological systems. For a valid qPCR result, suitable reference genes are needed in order to normalize the experiments, and ensure accuracy of the results. An ideal reference gene should be stably expressed in different plant tissues or developmental stages as well as under varying processing conditions. Blind selection of unverified or unstable reference genes has been shown to lead to inaccurate experimental results [27]. 

In this study, *UBC* and *TUB* showed the most constant level of expression and were considered the most suitable reference genes, while *ACTIN* is not recommended. Previous studies have demonstrated that *ACTIN* is unstable in different species and experimental conditions, such as in potato and longan [28,29]. However, *ACTIN* has also been described as the most stable gene in carrot [30]. Our findings showed that *UBC* was the best reference gene at different stages of development, which is in line with other studies [31]. In addition, *UBQ* has been reported to be the most stable gene in tomato [32], but not in rice [33] and celery [34]. Our findings further revealed stable expression levels of *TUB* consistent with what has been reported in carrot [30]. Conversely, this gene is not stably expressed in tea [35]. From our findings, we noted that *EF-1α* is not suitable for mustard stem unlike previous reports that have recommended it in wheat [36]. The stability of reference genes varies between species, tissues, developmental stages, diseases and infections, stresses, etc. [26]. Taken together, these results indicate that selecting a suitable reference gene is a very critical part of the expression analysis and provide a basis for future research on the mining, expression pattern, and regulatory mechanism of development-related functional genes. 

In plants, the XTH enzymes are widely found in various cells and tissues where they mainly catalyze cleavage and reconnection of xyloglucan molecules. Reports indicate that these genes relax cell wall in the process of cell growth and are therefore considered a key enzyme factor in the regulation of cell wall ductility [37]. So far, many XTH genes have been identified in plants, including 33 in *Arabidopsis* [38], 29 in rice [39], 61 in soybean [40], and 56 in tomato [41]. In the current study, we identified 74 XTH genes in *B.* *juncea* genome. Comparative genome studies have confirmed that the whole-genome triplication happened in evolution of *Brassica* species since its divergence from the *Arabidopsis* [42,43]. Based on the “U-triangle” model [44] and the hypothesis of *Brassica* triplication, the ratio between the number of *XTH* genes in *B.* *juncea* and *Arabidopsis* should be 6:1. In fact, the number of *XTH* genes in *B.* *juncea* was about 2.2 times than *Arabidopsis*. This may be due to the *Brassica* species have experienced a shrinking process featured by gene loss, fragmentation, and chromosomal rearrangement after polyploidy [43]. Several studies revealed that the orthologous numbers of some genes in *Brassica* crops and *Arabidopsis* were not completely conformed to triplication theory [45,46].

Various studies have analyzed the expression of *XTH* genes and reported their importance in a number of plant species. For instance, *XTH9* is important in plant growth and development in *Arabidopsis* [12], and when engineered into onion epidermis, it led to a significant cell wall elongation [47]. *XTH9* was also found to promote degradation of xyloglucan and increase ductility of hypocotyl cells in red beans [48]. Overexpressing *FvXTH9* and *FvXTH6* in strawberry accelerated fruit ripening [49], while in cotton, transgenic plants overexpressing *GhXTH1* produced 15 to 20 percent longer mature cotton fibers than wild types [50]. *AtXTH4* and *AtXTH24* were expressed highly in the hypocotyl of *Arabidopsis* [51], while *AtXTH9*, *AtXTH27,* and *AtXTH32* were responsible for stem elongation [52]. Genes with higher sequence homology seem to exhibit a similarity in function. *BjuB033943* and *BjuA012903*, which clustered with *AtXTH4* in Class I, maintained higher expression levels in stem development especially in the early stages. In Class II, high expression was observed in *BjuB008817*, *BjuA010942,* and *BjuA031509*, which clustered with *AtXTH9* clustered in adjacent branches. In addition, *BjuB040632* and *BjuA003577* with the closest evolution to *AtXTH24* were differentially expressed during development stages and maintained a high level of expression. Genes that showed the highest expression in Class III were *BjuA046283*, *BjuA046350*, *BjuB027461*, and *BjuA008769*. They also had relatively high homology compared with *AtXTH27*. The other four genes with high homology to *AtXTH32*, *BjuA036491*, *BjuA018261*, *BjuA010349,* and *BjuB016331* showed high expression level in S1 and S2 while their expression in S3 and S4 was downregulated. Results from the transcriptome and qPCR analysis showed that *XTH* genes expressed differentially during the period of stem development, indicating that they were extensively involved in stem swelling.

## Figures and Tables

**Table 1:** Information of candidate reference gene and qPCR amplification characteristics.

Gene Symbol	Gene Name	Primer Sequence (5’-3’)	Amplicon Size (bp)	Correlation Coefficient
*EF-1α*	Elongation factor -1α gene	CGTCTGCTTAGTGAACCTGCTC/ GAAGGAGCGAATGTCACAACCA	112	0.996
*HIS*	Histone	AGGGAAAGCCGCTCCACTTC/ TCGTAACCCTCTTGGCGTGAAT	152	0.996
*IF-4α*	Eukaryotic initiation factor 4 alpha	CAAGCCGAGCCTGCGATCAT/ TCGTGTTCTGGTCCATGTCTCC	121	0.996
*PP2A*	Protein phosphatase 2A	AAGCCGAGCCTTCCATCATAGC/ ACCACCACCACCACTCATTGC	197	0.992
*TUB*	Tubulin gene	GCGTCTTGTCCGTGAGATTGC/ GCCGAGATGAGGTGGTTAAGGT	109	0.994
*UBC*	Ubiquitin C gene	GCCATCACTCAGAGCGTCATCT/ AAGGAGACTGTGTAGGACCAAGAA	100	0.999
*UBQ*	Polyubiquitin 10 gene	TGCGTCTACCACTTCAGGATGT/ TCTGCTGGTCTGGAGGAATGC	145	0.992
*ACTIN*	Actin gene	ATCGTCTGTGACAACGGTAC/ ATGGAGGGTGATGAGATTCAGC	267	0.992

**Table 2:** Gene expression stability ranking of geNorm, NormFinder and BestKeeper.

Group	Rank	geNorm		NormFinder		BestKeeper		
		Gene	Stability	Gene Name	Stability Value	Gene	SD [±Cq]	CV [%Cq]
Total	1	*UBC*	0.49	*UBC*	0.31	*TUB*	0.67	2.73
	2	*UBQ*	0.49	*TUB*	0.39	*UBC*	0.77	3.02
	3	*TUB*	0.71	*UBQ*	0.48	*UBQ*	0.8	3.74
	4	*HIS*	0.92	*HIS*	0.59	*ACTIN*	0.86	2.91
	5	*EF-1α*	1.03	*IF-4α*	0.68	*HIS*	0.87	3.51
	6	*IF-4α*	1.06	*EF-1α*	0.7	*EF-1α*	0.9	4.01
	7	*PP2A*	1.22	*PP2A*	0.97	*IF-4α*	0.92	3.83
	8	*ACTIN*	1.36	*ACTIN*	1.06	*PP2A*	1	3.53
S1	1	*UBC*	0.33	*UBC*	0.11	*EF-1α*	0.28	1.26
	2	*TUB*	0.33	*IF-4α*	0.17	*IF-4α*	0.39	1.61
	3	*UBQ*	0.45	*TUB*	0.26	*HIS*	0.53	2.13
	4	*IF-4α*	0.52	*UBQ*	0.45	*PP2A*	0.66	2.26
	5	*PP2A*	0.61	*EF-1α*	0.45	*UBC*	0.71	2.67
	6	*EF-1α*	0.69	*PP2A*	0.45	*TUB*	0.81	3.29
	7	*HIS*	0.73	*HIS*	0.56	*UBQ*	0.9	4.01
	8	*ACTIN*	1.15	*ACTIN*	1.65	*ACTIN*	1.83	6.05
S2	1	*UBC*	0.26	*IF-4α*	0.19	*TUB*	0.25	1.02
	2	*HIS*	0.26	*UBC*	0.23	*IF-4α*	0.62	2.78
	3	*EF-1α*	0.37	*UBQ*	0.25	*ACTIN*	0.7	2.39
	4	*UBQ*	0.44	*EF-1α*	0.35	*UBQ*	0.86	4.09
	5	*IF-4α*	0.5	*TUB*	0.39	*UBC*	0.93	3.75
	6	*TUB*	0.58	*HIS*	0.42	*EF-1α*	1.05	4.9
	7	*PP2A*	0.71	*PP2A*	0.62	*HIS*	1.05	4.37
	8	*ACTIN*	0.88	*ACTIN*	0.93	*PP2A*	1.14	3.89
S3	1	*UBC*	0.4	*EF-1α*	0.11	*EF-1α*	0.23	0.96
	2	*TUB*	0.4	*UBC*	0.27	*UBC*	0.36	1.4
	3	*UBQ*	0.43	*PP2A*	0.33	*PP2A*	0.5	1.85
	4	*EF-1α*	0.48	*IF-4α*	0.38	*UBQ*	0.51	2.39
	5	*IF-4α*	0.61	*UBQ*	0.46	*TUB*	0.6	2.37
	6	*PP2A*	0.68	*TUB*	0.55	*IF-4α*	0.62	2.54
	7	*HIS*	0.82	*HIS*	0.68	*HIS*	0.73	2.84
	8	*ACTIN*	0.9	*ACTIN*	0.68	*ACTIN*	0.84	2.84
S4	1	*UBC*	0.25	*UBC*	0.08	*EF-1α*	0.38	1.68
	2	*TUB*	0.25	*TUB*	0.23	*PP2A*	0.57	2.09
	3	*IF-4α*	0.32	*EF-1α*	0.27	*IF-4α*	0.58	2.35
	4	*UBQ*	0.4	*IF-4α*	0.32	*UBC*	0.6	2.39
	5	*EF-1α*	0.48	*PP2A*	0.37	*TUB*	0.68	2.81
	6	*PP2A*	0.58	*UBQ*	0.37	*UBQ*	0.83	3.99
	7	*HIS*	0.65	*HIS*	0.55	*HIS*	0.94	3.83
	8	*ACTIN*	0.86	*ACTIN*	0.96	*ACTIN*	1.05	3.61

## Supplementary Materials

The following are available online at https://www.mdpi.com/2073-4425/11/1/113/s1. Figure S1: Amplification length of eight genes. Figure S2: Eight genes were amplified by qPCR using specific primers. Figure S3: Standard curve of eight candidate reference genes of *B. juncea*. Figure S4: The information of Cq values of the eight genes, Table S1: Candidate reference gene information and the primer sequences of cloning. Table S2: Cq value of candidate reference gene in four stem stages of stem mustard. Table S3: Determination of the optimal number of reference genes.
