# Supplementary material for: Expression Analysis of XTH in Stem Swelling of Stem Mustard and Selection of Reference Genes
Source: Genes (Basel). 2020 Jan 20;11(1):113. doi: 10.3390/genes11010113 (PMC7016721; doi:10.3390/genes11010113)
Supplement: Supplementary file 1 [file genes-11-00113-s001.zip › genes-653213-Supplementary-Figures.docx]

Supplementary Figures


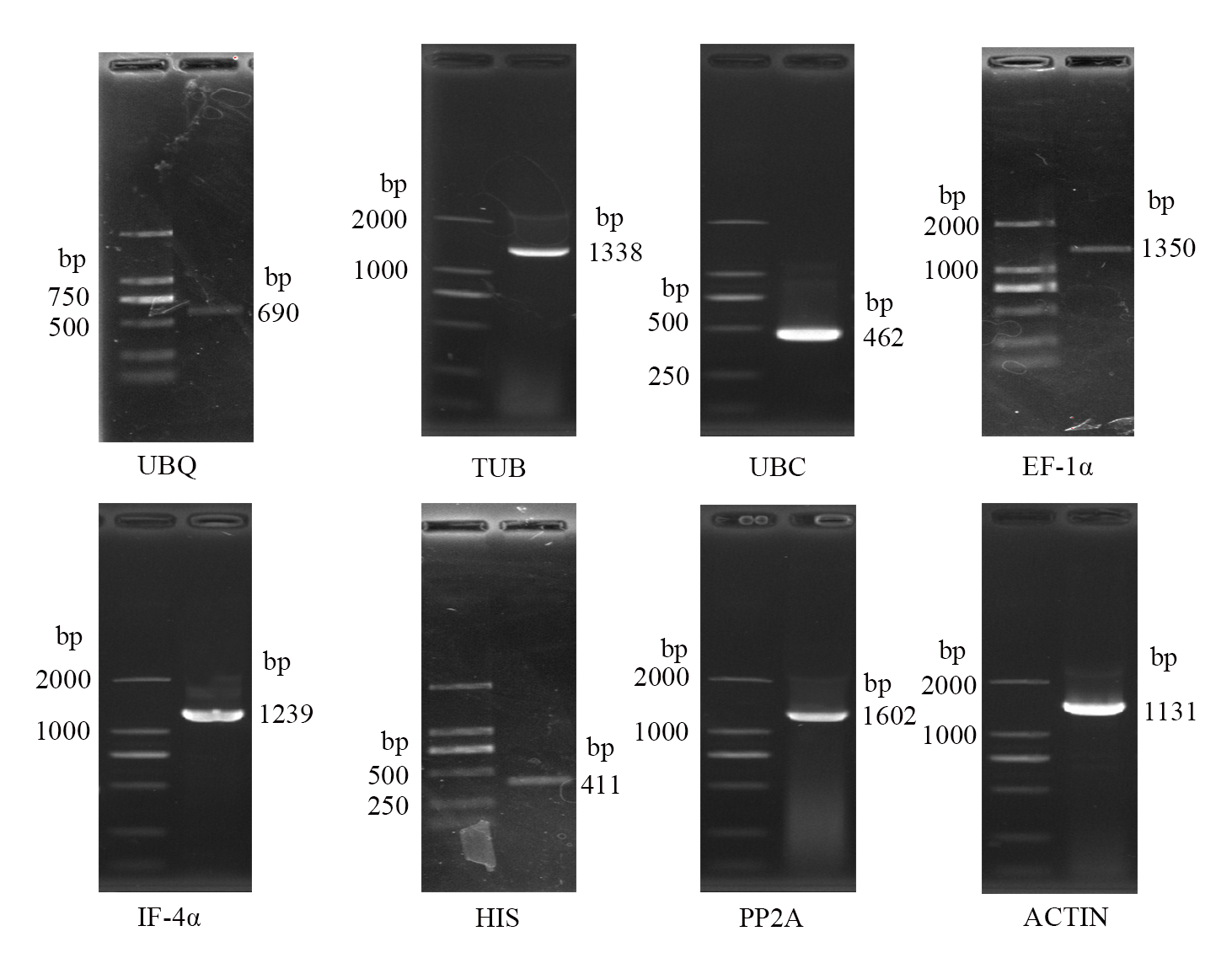


**Figure 1.** Amplification length of eight genes.


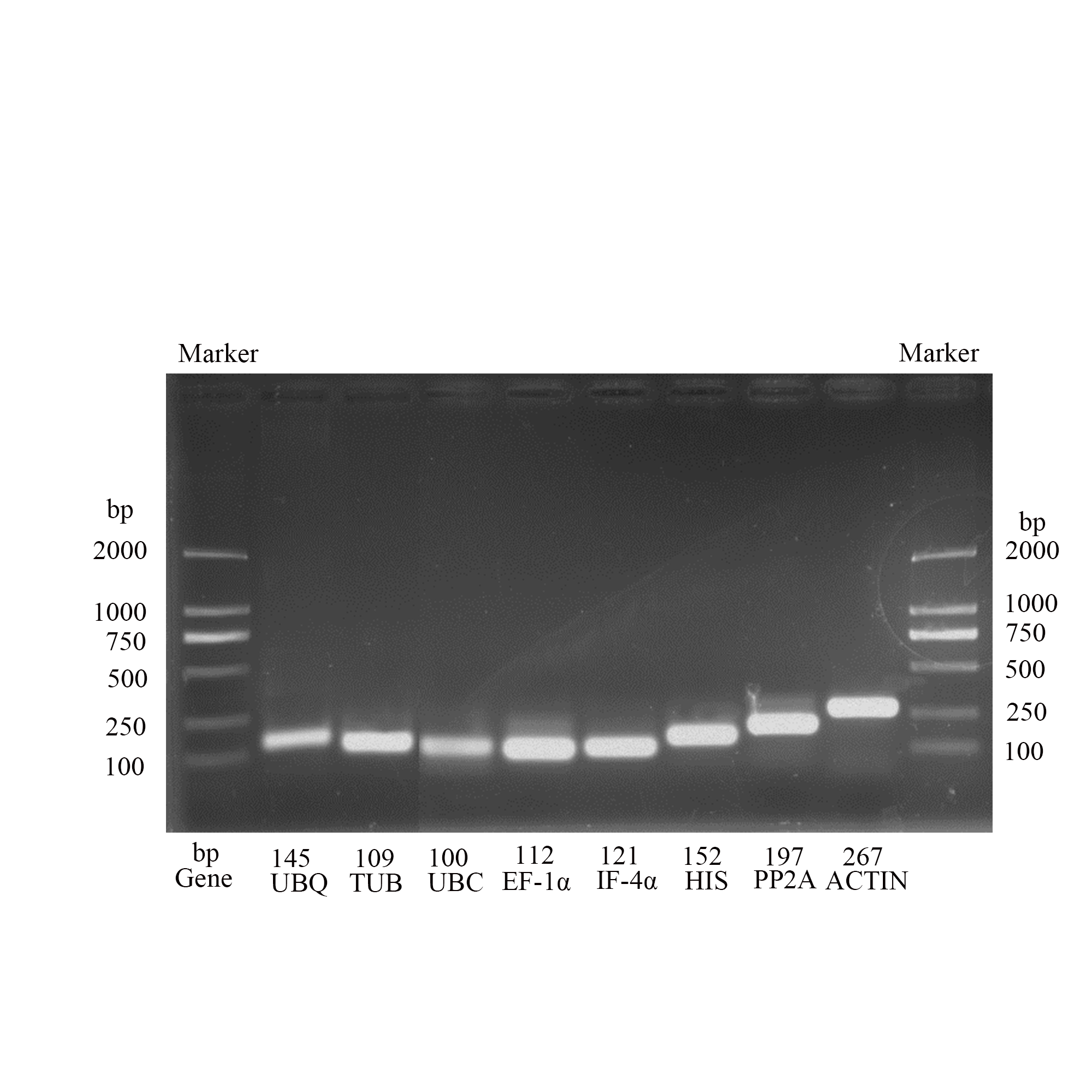


**Figure 2.** Eight genes were amplified by qPCR using specific primers.


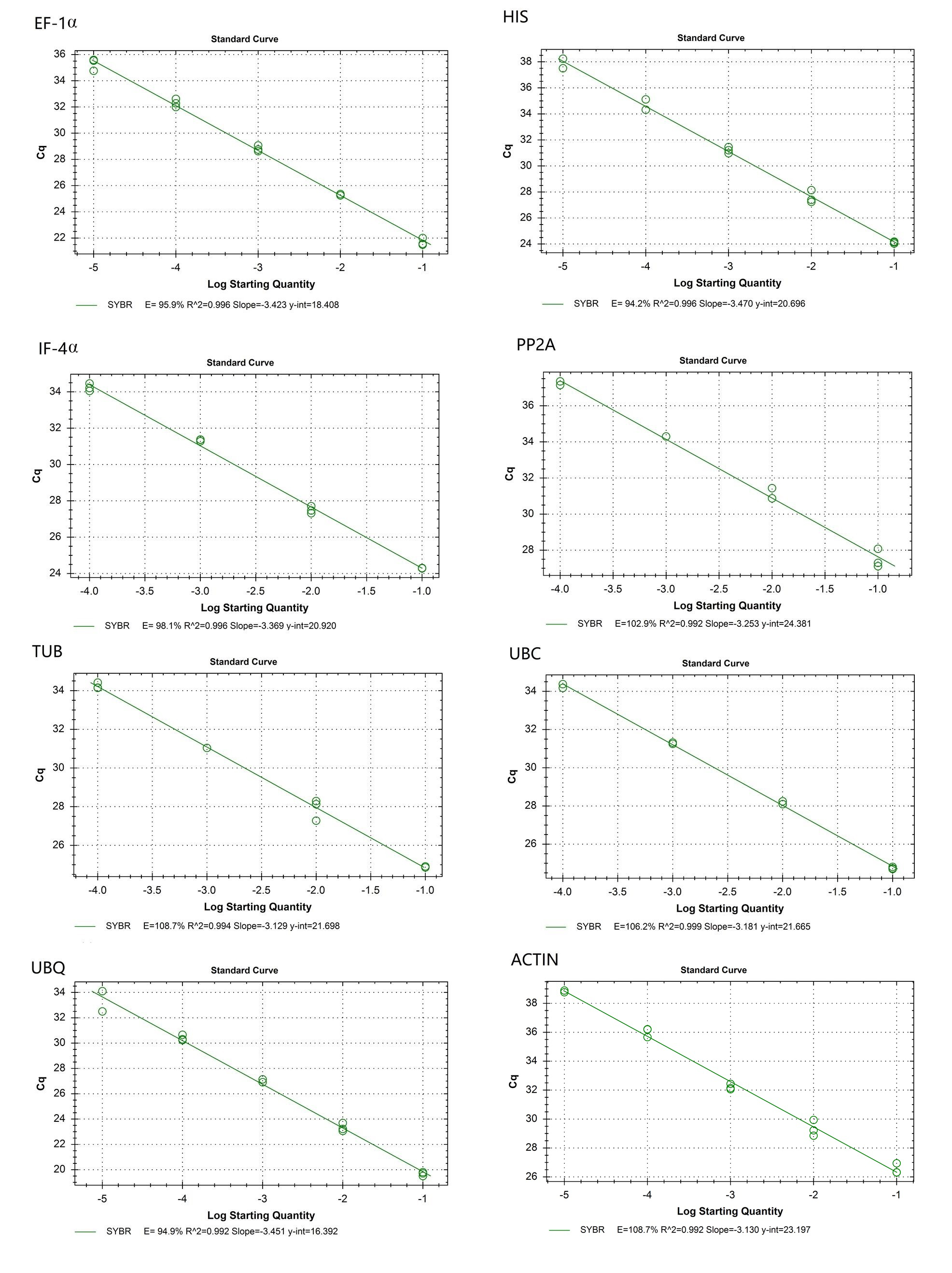


**Figure 3.** Standard curve of eight candidate reference genes of *B. juncea.*


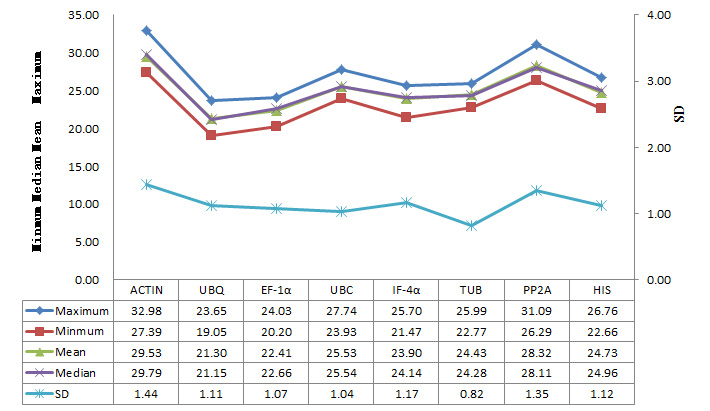


**Figure 4.** The information of Cq values of eight genes.
