# Supplementary material for: Expression Analysis of XTH in Stem Swelling of Stem Mustard and Selection of Reference Genes
Source: Genes (Basel). 2020 Jan 20;11(1):113. doi: 10.3390/genes11010113 (PMC7016721; doi:10.3390/genes11010113)
Supplement: Supplementary file 1 [file genes-11-00113-s001.zip › genes-653213-Supplementary-Tables.docx]

| **Gene symbol** | **Gene name** | **Primer sequence (5’-3’)** | **Amplicon size (bp)** |
| --- | --- | --- | --- |
| EF-1α | Elongation factor -1α gene | ATGGGTAAAGAGAAGTTTCACA/  TCATTTGGCACCCTTCTTGAC | 1350 |
| HIS | Histone | ATGGCTCGTACCAAGCAAA/  TCAAGCTCTTTCACCACGG | 411 |
| IF-4α | Eukaryotic initiation factor 4 alpha | ATGGCAGGATCCGCACC/  TCAAAGCAGATCAGCAACGT | 1239 |
| PP2A | Protein phosphatase 2A | ATGTCGATGGTCGAAGAGC/  TAGCTAGACATCATCACGTTATC | 1602 |
| TUB-B | β-[Tubulin](http://link.springer.com/search?dc.title=Tubulin&facet-content-type=ReferenceWorkEntry&sortOrder=relevance) gene | ATGAGAGAGATCCTCCACATC/  TCAAGCCTCATCGTATTCCTC | 1338 |
| UBC | Ubiquitin C gene | ATGGCGAATAGCAATCTACC/  TCAAGCGCCACTTGCATATA | 462 |
| UBQ | Polyubiquitin 10 gene | ATGCAGATCTTCGTTAAAACAC/  CCACCACGGAGACGCAA | 690 |
| ACTIN | Actin gene | ATGGAGGGTGATGAGATTCAGC/  TTAGAAACATTTTCTGTGAACAATC | 1,131 |

**Table 1.** candidate reference gene information and the primer sequences of cloning.

**Table 2.** Cq value of candidate reference gene in four stem stages of stem mustard.

|  | **Stage** | ***ACTIN*** | ***UBQ*** | ***EF-1α*** | ***UBC*** | ***IF-4α*** | ***TUB*** | ***PP2A*** | ***HIS*** |
| --- | --- | --- | --- | --- | --- | --- | --- | --- | --- |
| S1 | 1-1 | 27.392 | 22.79 | 22.22 | 26.61 | 24.36 | 24.69 | 29.34 | 25.24 |
|  | 1-1 | 27.514 | 22.80 | 22.19 | 26.85 | 24.27 | 25.02 | 30.99 | 25.44 |
|  | 1-1 | 27.409 | 23.65 | 22.35 | 26.69 | 24.33 | 25.02 | 29.11 | 25.61 |
|  | 1-2 | 32.160 | 23.13 | 21.66 | 27.74 | 24.38 | 25.22 | 29.29 | 24.52 |
|  | 1-2 | 32.980 | 23.43 | 21.59 | 27.66 | 24.32 | 25.35 | 30.15 | 24.55 |
|  | 1-2 | 32.415 | 22.93 | 21.64 | 27.74 | 24.84 | 25.78 | 29.73 | 24.59 |
|  | 1-3 | 30.232 | 21.15 | 21.41 | 25.62 | 23.45 | 23.26 | 28.64 | 24.08 |
|  | 1-3 | 30.942 | 20.68 | 21.68 | 25.46 | 23.60 | 23.51 | 27.72 | 24.14 |
|  | 1-3 | 30.560 | 21.46 | 21.82 | 25.90 | 23.58 | 23.31 | 28.81 | 25.51 |
| S2 | 2-1 | 28.892 | 21.76 | 22.85 | 26.24 | 23.08 | 24.18 | 30.25 | 25.78 |
|  | 2-1 | 29.763 | 22.16 | 22.81 | 26.20 | 23.35 | 24.59 | 31.09 | 25.13 |
|  | 2-1 | 28.532 | 21.96 | 23.14 | 26.54 | 23.12 | 24.34 | 30.96 | 25.53 |
|  | 2-2 | 29.686 | 20.14 | 20.20 | 24.29 | 21.47 | 23.89 | 27.28 | 23.22 |
|  | 2-2 | 28.933 | 20.16 | 20.26 | 24.22 | 21.58 | 23.93 | 29.92 | 23.25 |
|  | 2-2 | 29.149 | 21.69 | 20.72 | 24.54 | 21.56 | 24.22 | 29.03 | 24.03 |
|  | 2-3 | 27.590 | 20.07 | 20.58 | 24.03 | 21.94 | 23.64 | 27.40 | 22.87 |
|  | 2-3 | 29.870 | 20.23 | 20.76 | 24.09 | 22.02 | 23.79 | 29.61 | 22.66 |
|  | 2-3 | 30.830 | 20.19 | 20.96 | 24.16 | 22.19 | 23.92 | 28.51 | 22.98 |
| S3 | 3-1 | 29.918 | 20.59 | 23.86 | 25.67 | 25.31 | 25.20 | 27.14 | 24.97 |
|  | 3-1 | 29.508 | 20.96 | 23.76 | 25.79 | 25.37 | 25.35 | 27.59 | 24.98 |
|  | 3-1 | 30.374 | 21.99 | 24.03 | 25.89 | 25.32 | 25.82 | 28.03 | 25.24 |
|  | 3-2 | 28.064 | 21.43 | 23.35 | 25.30 | 23.48 | 25.44 | 26.29 | 24.94 |
|  | 3-2 | 27.476 | 21.79 | 23.32 | 25.76 | 23.51 | 25.46 | 26.80 | 25.20 |
|  | 3-2 | 30.334 | 21.62 | 23.45 | 25.68 | 24.06 | 25.99 | 26.75 | 25.05 |
|  | 3-3 | 30.131 | 20.37 | 23.40 | 24.82 | 24.09 | 24.23 | 27.37 | 26.76 |
|  | 3-3 | 30.390 | 20.46 | 23.23 | 25.00 | 24.19 | 24.23 | 27.19 | 26.69 |
|  | 3-3 | 30.283 | 21.04 | 23.48 | 25.02 | 24.31 | 24.15 | 28.43 | 26.65 |
| S4 | 4-1 | 28.147 | 20.89 | 22.62 | 25.43 | 24.52 | 24.15 | 28.08 | 25.66 |
|  | 4-1 | 28.759 | 21.05 | 22.70 | 25.41 | 24.69 | 24.11 | 28.13 | 25.89 |
|  | 4-1 | 30.554 | 21.50 | 23.31 | 25.73 | 25.48 | 24.69 | 28.22 | 26.06 |
|  | 4-2 | 28.570 | 20.82 | 23.11 | 25.19 | 25.09 | 24.43 | 27.60 | 24.55 |
|  | 4-2 | 27.510 | 21.15 | 23.35 | 25.38 | 25.56 | 24.76 | 27.12 | 24.58 |
|  | 4-2 | 29.950 | 22.47 | 23.33 | 25.84 | 25.70 | 24.98 | 26.88 | 24.69 |
|  | 4-3 | 29.814 | 19.05 | 22.27 | 23.93 | 23.72 | 22.77 | 26.65 | 22.91 |
|  | 4-3 | 27.775 | 19.28 | 22.31 | 24.13 | 24.54 | 23.11 | 26.41 | 23.04 |
|  | 4-3 | 30.806 | 20.03 | 23.15 | 24.39 | 23.91 | 23.13 | 27.20 | 23.43 |

**Table 3.** Determination of the optimal number of reference genes.

| **geNorm** | **Total** | **S1** | **S2** | **S3** | **S4** |
| --- | --- | --- | --- | --- | --- |
| V2/3 | 0.261321 | 0.163227 | 0.136665 | 0.134044 | 0.111747 |
| V3/4 | 0.264653 | 0.131442 | 0.117377 | 0.119523 | 0.115901 |
| V4/5 | 0.212533 | 0.13462 | 0.106916 | 0.153671 | 0.109436 |
| V5/6 | 0.151965 | 0.127782 | 0.110508 | 0.124581 | 0.119011 |
| V6/7 | 0.212601 | 0.103927 | 0.136981 | 0.15521 | 0.108307 |
| V7/8 | 0.195883 | 0.298025 | 0.166436 | 0.12677 | 0.175442 |
